# Normalisation of the psychometric encephalopathy score within the Cameroonian population

**DOI:** 10.1186/s12876-021-01858-7

**Published:** 2021-07-12

**Authors:** Larissa Pessidjo Djomatcho, Mathurin Pierre Kowo, Antonin Ndjitoyap Ndam, Sylvain Raoul Simeni Njonnou, Gabin Ulrich Kenfack, Firmin Ankouane Andoulo, Servais Fiacre Eloumou Bagnaka, Winnie Tatiana Bekolo, Agnès Malongue, Isabelle Dang Babagna, Magloire Biwolé Sida, Henry Luma, Oudou Njoya

**Affiliations:** 1 Faculty of Medicine and Biomedical Sciences, University of Yaoundé I, Yaoundé, Cameroon; 2 University Teaching Hospital Yaounde, Yaoundé, Cameroon; 3 Yaounde General Hospital, Yaoundé, Cameroon; 4 Faculty of Medicine and Pharmaceutical Sciences, University of Dschang, Dschang, Cameroon; 5 Yaounde Central Hospital, Yaoundé, Cameroon; 6 Faculty of Medicine and Pharmaceutical Sciences, University of Douala, Douala, Cameroon; 7 Gynaeco-Obstetric and Paediatric Hospital Douala, Douala, Cameroon; 8 Douala General Hospital, Douala, Cameroon; 9 Cathedral Medical Centre Yaounde, Yaoundé, Cameroon

**Keywords:** Cirrhosis, Minimal hepatic encephalopathy, Psychometric tests, Sub-Saharan Africa, Cameroon

## Abstract

**Background:**

Minimal hepatic encephalopathy (MHE) is the presence of neuropsychological abnormalities detectable by psychometric tests. Psychometric Hepatic Encephalopathy Score (PHES) is a gold standard test for the early diagnosis of MHE in cirrhotic patients. The aim of this study was to standardize the PHES in a healthy Cameroonian population and to evaluate the prevalence of MHE among cirrhotic patients.

**Methods:**

This was a prospective, multicentric study from 1 December 2018 to 31 July 2019 in two groups: healthy volunteers and cirrhotic patients without clinical signs of hepatic encephalopathy. The results of the number connection test-A, number connection test-B, serial dotting test, line tracing test were expressed in seconds and those of the digit symbol test in points.

**Results:**

A total of 102 healthy volunteers (54 men, 48 women) and 50 cirrhotic patients (29 men, 31 women) were included. The mean age was 38.1 ± 12.55 years in healthy volunteers and 49.3 ± 15.6 years in cirrhotic patients. The mean years of education level was 11.63 ± 4.20 years in healthy volunteers and 9.62 ± 3.9 years in cirrhotic patients. The PHES of the healthy volunteer group was − 0.08 ± 1.28 and the cut-off between normal and pathological values was set at − 3 points. PHES of the cirrhotic patients was − 7.66 ± 5.62 points and significantly lower than that of volunteers (*p* < 0.001). Prevalence of MHE was 74% among cirrhotic patients. Age and education level were associated with MHE.

**Conclusion:**

PHES cut-off value in Cameroonians is − 3, with MHE prevalence of 74% among cirrhotic patients.

**Supplementary Information:**

The online version contains supplementary material available at 10.1186/s12876-021-01858-7.

## Background

The notion of minimal hepatic encephalopathy (MHE) was developed for the first time in the 1970s when cirrhotic patients with normal physical examination had pathological neuropsychological tests [1–3]. MHE is the earliest form of hepatic encephalopathy characterised by neurocognitive, alertness and integrative function involvement [4]. Most often it is not diagnosed at the beginning due to its subclinical nature whereas it alters life quality, increases the risk of domestic and car accidents, predisposes to clinical hepatic encephalopathy and reduces life expectancy [2, 3, 5].

Psychometric Hepatic Encephalopathy Score (PHES) is the score of the porto-systemic encephalopathy (PSE) battery, which is made up of five (5) psychometrical tests. It is recommended as a first line diagnostic tool for MHE [1]. These include Digit Symbol Test (DST), Number connection Test-A (NCT-A), Number connection Test-B (NCT-B), Serial Dotting Test (SDT), Line tracing Test (LTT). They assess psychomotor retardation, attention deficit and executive functions [6]. The PHES is expressed as the number of standard deviation (SD) in a population matched with respect to age and educational level.

Studies on MHE in Sub-Saharan Africa are scarce. In Cameroon, MHE is an entity which is not well known and so, scarcely assessed. This study aimed at standardising the PHES in a healthy Cameroonian population and to evaluate the prevalence of MHE among Cameroonian patients with liver cirrhosis.

## Methods

This was a prospective, multicentric and cross-sectional study from 1 December 2018 to 31 July, 2019 in two case-studied populations. On the one hand, healthy volunteers and on the other hand, cirrhotic patients who did not show clinical signs of hepatic encephalopathy (HE). This study was carried out in 5 hospitals in the cities of Douala and Yaounde (Douala General Hospital, Gynaeco-Obstetric and Paediatric Hospital Douala, Yaounde Central Hospital, University Teaching Hospital Yaounde, Yaounde General Hospital). Illiterate participants were not involved as they could neither read nor write French or English. Participants with at-risk alcohol consumption (greater than 30 g/day in men and 20 g/day for women during the last three months), consuming lactulose, antibiotics or psychotropic drugs during the last few weeks prior to inclusion, with cognitive, psychiatric or neurological disorders or having visual disturbances not corrected by wearing glasses and patients who recently had a spontaneous infection of ascitic fluid or gastrointestinal bleeding or with co-morbidities such as: heart failure, renal failure or respiratory failure were also excluded.

### Group of healthy volunteers

Healthy volunteers were recruited among healthy ordinary Cameroonians, most often family members of patients who accompanied them for medical consultation.

Persons with a risky alcohol consumption in the last three months (> 30 g/day in males and > 20 g/day in females), chronic liver disease, psychiatric or neurologic disease, renal failure, heart failure, use of psychotic drugs, were excluded from this group. For each volunteer, socio-demographic data (age, gender, level of education) and past medical history were recorded. The volunteers were grouped by age (20–29, 30–39, 40–49, 50–59, > 60 years), gender and level of education (less than or equal to 5 years, 6–12 years, > 12 years).

### Group of cirrhotic patients

The diagnosis of cirrhosis was based on clinical, biological, morphological and endoscopic evidence of portal hypertension and liver failure or elastography criteria (Fibrosis score 4). For each cirrhotic patient, socio-demographic data (age, gender, level of education), past history (alcohol consumption, smoking, past history of hepatic encephalopathy, gastrointestinal bleeding, spontaneous bacteria peritonitis in the last three months, presence of hepatocellular carcinoma and psychiatric follow up) were recorded. Clinical data included signs of clinical hepatic encephalopathy: mental confusion, asterixis; signs of portal hypertension and chronic liver failure: jaundice, splenomegaly, hepatomegaly, ascites and pedal edema. Para-clinical data included complete blood count, serum albumin, total bilirubin, prothrombin time, international normalised ratio, etiology of cirrhosis, ultrasound evidence of cirrhosis, endoscopic evidence of portal hypertension (esophageal varices, gastric varices). Patients were classified either using Child or MELD (Model for End Stage Liver Disease) score [7].

### Psychometric tests

All healthy volunteers and cirrhotic patients had a series of 5 psychometric tests made up of:

1. NCT-A: It consists in connecting numbers in an arithmetic order (1–25) as quickly as possible. Time spent to complete the test and to correct any errors is expressed in seconds.
2. NCT-B: It consists in linking numbers and letters in an alternatively (1-A, 2-B, 3-C). Time taken to complete the test and time spent to correct any errors is expressed in seconds.
3. SDT: It consists in making dots at the centre of 10 circles as fast as possible from left to right. Time spent to complete the test and to correct any errors is expressed in seconds.
4. DST: It consists in linking symbols to corresponding numbers as quickly as possible. The time required to complete the test is 90 s. The result is expressed in points.
5. LTT: It consists in drawing a dash between 2 lines 5 mm apart without touching the borders as fast as possible. The LTT results is expressed in terms of time spent to complete the test (LTTt seconds) and error score (LTTe), LTT = (1 + LTTe/100) × LTTt (according to Italian standardisation) [8].

The tests were performed using a pencil. After an initial demonstration, participants performed the test following recommendations in a calm and lighted place between 8 AM to 3 PM.

The results of cirrhotic patients were compared to those of healthy volunteers with the same age and educational level. Test results of (NCT-A, NCT-B, SDT, LTT) found between the mean ± 1 standard deviation (SD) were given a score of 0 points. Those between the mean, + 1SD and + 2SD were given a score of − 1 point, those between the mean + 2SD and + 3SD were given a score of − 2 points, those above the mean + 3SD were given a score of − 3. Test results with a mean less than − 1SD were given a score of + 1 points. SDT results found between the mean ± 1SD were given a score of 0 points. Test results found between the mean − 1SD and − 2SD, − 2 and − 3 SD and less than − 3SD were respectively given − 1, − 2, − 3 points. Test results greater than the mean + 1SD were given a score of + 1 point. PHES was the sum of these 5 tests expressed in terms of the number of standard deviations. Patients were classified as having MHE when their PHES was less than − 3.

### Statistical analysis

Data analysis was carried out via the Statistical Package for Social Sciences (SPSS Inc, Chicago, Illinois, USA) version 23.0. Depending on quantitative data distribution, they were expressed as mean ± SD or median and interquartile range. Categorical data were expressed as numbers and proportions. Means and medians were compared using the *t* test and the Mann–Whitney *U*-test respectively. Proportions were compared using the chi-squared test or Fisher’s exact test. Levene’s test was used to evaluate the differences in variance. Non-parametric tests were applied if homogeneity of variance assumptions were not met. The Shapiro–Wilk test was performed to test the hypothesis that the variables came from a normally distributed population. The regression equations were used to calculate scores for individual tests. Correlations were tested with Pearson’s r or Spearman’s R, where applicable. A two-sided *p* value < 0.05 was considered significant.

## Results

### Standardisation sample

One hundred and two (102) healthy volunteers were enrolled, including 54 males (52.90%) and 48 females (48.10%). The mean age was 38.1 ± 12.55 years (range 20–72 years). The mean number of years of study was 11.63 ± 4.20 years (range 4–22 years). Data are summarised in Table 1.

**Table 1:** Distribution of volunteers (n = 102) in relation to age groups

Age	Gender (male/female)	Education (years, mean ± SD, range)
[20–30] years (n = 29)	18 (62.1%)/11 (37.9%)	12 ± 4 (7–21)
[30–40] years (n = 31)	16 (51.6%)/15 (48.4%)	13 ± 5 (4–22)
[40–50] years (n = 19)	8 (42.1%)/11 (57.9%)	11 ± 3 (5–16)
[50–60] years (n = 17)	9 (52.9%)/8 (47.1%)	10 ± 4 (4–15)
[60–72] years (n = 6)	3 (50%)/3 (50%)	6 ± 2 (4–6)

The results of NCT-A, NCT-B, SDT, DST, LTT tests were respectively 85.14 ± 30.78 s; 120.33 ± 64.63 s; 73.56 ± 20.55 s; 34.47 ± 10.40 points; 109.83 ± 33.93 s.

Age and education were significantly correlated with all test performance except for LTT with age (Table 2).

**Table 2:** Correlations between single psychometric tests results and PHES and studied variables in standardization sample subjects (n = 102)

	Age (years)	Education (years)
NCT-A	r = 0.30 (*p* = 0.002)	r = − 0.67 (*p* < 0.001)
NCT-B	r = 0.33 (*p* = 0.001)	r = − 0.70 (*p* < 0.001)
DST	r = − 0.50 (*p* < 0.001)	r = 0.71 (*p* < 0.001)
LTT	r = 0.17 (*p* = 0.08)	r = − 0.44 (*p* < 0.001)
SDT	r = 0.29 (*p* = 0.003)	r = − 0.42 (*p* < 0.001)
PHES	r = − 0.49 (*p* < 0.001)	r = 0.70 (*p* < 0.001)

*NCT-A* Number connection test-A, *NCT-B* number connection test-B, *DST* digit symbol test, *LTT* line tracing test, *SDT* serial dotting test, *PHES* psychometric hepatic encephalopathy score

After multivariate analysis, predictive equations were parameterised on age and education to compute the standard deviations and scores for each test (Table 3).

**Table 3:** Predictors of psychometric tests on multivariate analysis (multiple regression models)

Test	Equation	SD
NCT-A	125.62 + 0.3 × age − 4.47 × education	11.69
NCT-B	159.82 + 1.39 × age − 7.96 × education	24.65
SDT	78.89 + 0.35 × age − 1.6 × education	9.39
LTT	150.02 + 0.11 × age − 3.82 × education	15.10
DST	23.40 − 0.20 × age + 1.63 × education	3.24

Age and education are expressed in years

*NCT-A* number connection test-A, *NCT-B* number connection test-B, *SDT* serial dotting test, *LTT* line tracing test, *DST* digit symbol test, *SD* standard deviation

The mean PHES score in volunteers was − 0.08 ± 1.28 (range − 3 to + 4) points. The limit between normal and pathologic PHES was calculated at mean-2SD and was set at − 3 points. MHE was diagnosed at PHES score less than − 3 points. In the healthy volunteer group, the score PHES was correlated with education years and age (Table 2). There was no association between each test or PHES score with gender (*p* ≥ 0.05).

### PHES results in patients with cirrhosis

We recruited 70 cirrhotic patients among which 20 were excluded due to one or more exclusion criteria; illiterate (10), hepatocellular carcinoma (6), visual impairments (4). Fifty patients without clinical evidence of HE were retained including 29 males (58%) and 21 females (42%). The characteristics of cirrhotic patients are described in Table 4.

**Table 4:** Characteristics of cirrhotic patients

Variables	Patients with cirrhosis
Age (years, mean ± SD, range)	49.3 ± 15.6 [21–78] (n = 50)
Gender (male/female)	29 (58%)/21 (42%)
Education (years, mean ± SD, range)	9.62 ± 3.9 (2–19) (n = 50)
Aetiology of cirrhosis	
Viral hepatitis B	33 (66%)
Viral hepatitis C	17 (34%)
Child–Pugh class	
A	26 (60.5%)
B	12 (27.9%)
C	5 (11.6%)
MELD score (score, mean ± SD, range)	10.7 ± 3.5 (6–18) (n = 22)
Ascites (yes/no)	17 (34%)/33 (66%)
Jaundice (yes/no)	9 (18%)/41 (82%)
Previous spontaneous bacterial peritonitis (yes/no)	1 (2%)/49 (98%)
Previous overt HE (yes/no)	1 (2%)/49 (98%)
Previous GI tract hemorrhage (yes/no)	8 (16%)/42 (84%)
Creatinine (mg/dl, mean ± SD, range)	9.1 ± 3 (3–17.5) (n = 42)
INR (Median, IQR)	1.19 [1.0–1.8] (n = 20)
Albumin (g/l, mean ± SD, range)	34.4 ± 7 (19–45) (n = 26)
Bilirubin (mg/dl, Median, IQR)	9.4 [1.1–68.0] (n = 31)
ALT (IU/L, Median, IQR)	35.5 [11–155] (n = 32)

*MELD* model of end-stage liver disease, *HE* hepatic encephalopathy

**Fig. 1:**
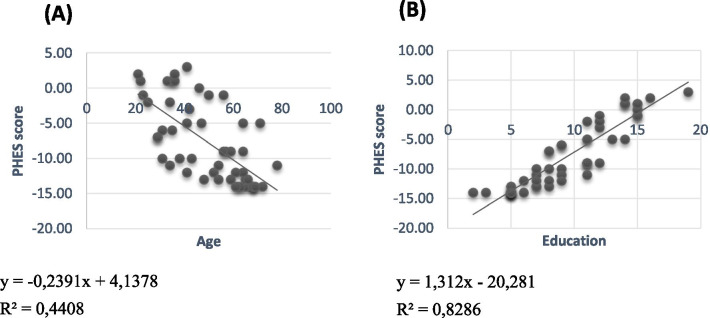
Correlations between psychometric hepatic encephalopathy score (PHES) and age and education in patients with cirrhosis: age (**a**) and education (**b**)

NCT-A, NCT-B, SDT, DST, LTT means values were respectively 164.72 ± 169.93 s; 255.96 ± 203.69 s; 150.26 ± 93.26 s; 22.62 ± 11.36 points; 183.03 ± 134.54 s. The mean PHES in the cirrhotic group was − 7.66 ± 5.62 (range − 14 to + 3). Thus, we had a prevalence of 74% (37/50) for minimal hepatic encephalopathy in cirrhotic patients. Patients with abnormal PHES score were older and less educated than normal subjects (Table 5 and Fig. 1). No significant correlation between PHES score and biological parameters was observed: Alanine aminotransferase (ALAT), bilirubin, prothrombin time, albumin, creatinine, International Normalized Ratio (INR), platelets (*p* ≥ 0.05). Comparisons of clinical and biochemical data of patients with normal and pathological PHES is presented in Table 5. We found significant difference in PHES score between cirrhotic patients and normal participants (*p* < 0.001).

**Table 5:** Clinical and biochemical data of cirrhotic patients with MHE and no MHE

Demographic data	MHE	No MHE	OR [95% CI]	*p* value
Age (Years, mean ± SD)	54.3 ± 13.9 (n = 37)	35.2 ± 10.9 (n = 13)	–	< 0.001
Male gender	20 (54.1%)	9 (69.2%)	0.52 [0.1–2.0]	0.51
Education (Years, mean ± SD)	7.9 ± 2.8 (n = 37)	14.3 ± 2.0 (n = 13)	–	< 0.001
CHILD–PUGH B/C	15 (48.4%)	2 (16.7%)	4.68 [0.8–24.9]	0.08
A	16 (51.6%)	10 (83.3%)	1	1
Jaundice (Yes/no)	8 (21.6%)	1 (7.7%)	3.31 [0.3–29.4]	0.41
Ascites (Yes/no)	14 (37.8%)	3 (23.1%)	2.02 [0.4–8.6]	0.49
Previous GI hemorrhage (Yes/no)	6 (16.2%)	2 (15.4%)	1.06 [0.1–6.0]	1.00
MELD score (Score, mean ± SD)	11.6 ± 3.7 (n = 15)	8.8 ± 2.6 (n = 7)	–	0.09
INR (Median, IQR)	1.2 [1.0–1.8] (n = 13)	1.1 [1.0–1.6] (n = 7)	–	0.75
Albumin (g/l, mean ± SD)	33.8 ± 7.0 (n = 21)	36.8 ± 7.3 (n = 5)	–	0.41
Creatinin (mg/dl, mean ± SD)	9.4 ± 3.4 (n = 31)	8.5 ± 1.8 (n = 11)	–	0.40
Prothrombin time (%)	65.7 ± 18.7 (n = 30)	59.6 ± 13.7 (n = 10)	–	0.35
Bilirubin (mg/dl, Median, IQR)	8.7 [1.1–68.0] (n = 24)	10.0 [8.0–41.0] (n = 7)	–	0.14
ALAT (IU/l, Median, IQR)	33 [15–155] (n = 20)	46 [11–76] (n = 12)	–	0.34
Platelets (/ml, Median, IQR)	110.10^3^ [15.10^3^–421.10^3^] (n = 37)	91.10^3^ [42.10^3^–300.10^3^] (n = 12)	–	0.81

*MHE* minimal hepatic encephalopathy, *MELD* model for end stage liver disease, *INR* International normalized ratio, *ALAT* alanine aminotransferase

In patients with cirrhosis, we calculated PHES using the Spanish reference data (Additional file 1).

**Fig. 2:**
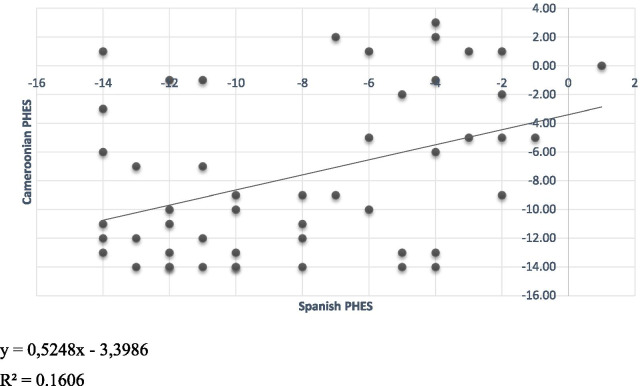
Relationship between psychometric hepatic encephalopathy score (PHES) calculated by Spanish and Cameroonian reference data in patients with cirrhosis (n = 50)

Using the online PHES score calculator for Spanish population available on www.redeh.org [9], the PHES score was calculated for each cirrhotic patient and compared with the obtained values with the values of Cameroonians. There was a correlation between the two scoring systems (r = 0.36; *p* = 0.01, Fig. 2).

## Discussion

This cross-sectional study aiming at standardising the PHES in a healthy Cameroonian population and to evaluate the prevalence of MHE among Cameroonian patients with liver cirrhosis showed that the PHES was significantly lower among cirrhotic patients than in healthy volunteers. In four cirrhotic patients nearly three had MHE, and age and education level were associated with MHE.

The sociodemographic and clinical characteristics of healthy volunteers and cirrhotic patients were similar to the findings reported in literature [10–12]. A male predominance could be related to a greater exposure to risk factors for cirrhosis such as alcohol consumption [13]. The observed variation of the level of education across studies could be explained by the sociocultural diversities of the various populations studied.

Previous studies reported that PHES is globally influenced by age and level of education [1]; the present findings are similar as they show that age and level of education were significantly correlated with all tests performance except for LTT with age; the latter could be explained by the younger age of the volunteers. The hypothesis on whether the young age of volunteers gives them greater speed in carrying out the LTT still needs to be proven.

The results of NCT-A, NCT-B, SDT, DST, LTT and PHES score were consistent with the results reported elsewhere revealing the influence of age and level of education on psychometric tests [10–12]. This could be explained by a decline in cognitive function with age and a certain degree of cognitive incapacity associated with low level of education [14]

There was a higher prevalence of MHE in this studied population occurring in 74% of cirrhotic patients compared to the result from the literature [5, 15, 16]. The reported higher prevalence in our study could be explained by the age, the low level of education and the presence of the signs of decompensated cirrhosis which were more important in patients with MHE.

In contrast with reports from the literature, the study shows no correlation between Child Pugh score [10, 11], MELD score [17], gender, aetiology of cirrhosis and the onset of MHE. In this study, the biological parameters for Child Pugh score calculation were not available for many cirrhotic patients. Child Pugh score could not be compared as a quantitative variable but as a qualitative variable distinguishing two groups (Child Pugh A on the one hand, Child Pugh B and C on the other hand) and that might explain the absence of association between Child–Pugh score and minimal hepatic encephalopathy. MELD score was equally available in less than half of cirrhotics patients and the absence of this parameter could explain the present findings. However, it was noticed that more than half of patients with MHE were Child Pugh A. This result underlines the fact that screening for MEH should also be done in Child Pugh A cirrhotic patients.

### Limitations

This study has some limitations.

The population of cirrhotic patients was small due to the numerous exclusion criteria.

Because of the high cost of laboratory tests and the absence of universal health coverage in our setting, all laboratory tests (serum ammonia levels) could not be carried out and thus affecting the quality of this study.

For many cirrhotic patients, biological parameters for Child Pugh score and MELD score calculation were not available. Child Pugh score could not be compared as a quantitative variable but as a qualitative variable distinguishing two groups (Child Pugh A on the one hand, Child Pugh B and C on the other hand) and this, associated with the reduced size of our cirrhotic population might explain the absence of association between Child–Pugh score and the minimal hepatic encephalopathy.

A further limit of the study is that the reference population was younger and with higher education than cirrhotic patients and this fact may have influenced cut-off values; even though tests were standardised for age and education. Further prospective studies will confirm the best cut-off for a diagnosis of MHE in Cameroon.

## Conclusion

The PHES was standardised in the Cameroonian population of healthy volunteers. This enabled to make the diagnosis of MHE in 74% of cirrhotic patients with no clinical evidence of encephalopathy. Age and education level were identified as factors associated with MHE of this study. Cirrhotic patients with Child Pugh A should be screened for the diagnosis of MHE. Further studies on larger cohorts have to be carried out to confirm these findings.

## Supplementary Information

**Additional file 1.** Example of calculation of the Z score for each test and the PHES of a cirrhotic patient.

## Data Availability

The datasets used during this study are available from the corresponding author on a reasonable request.

## Abbreviations

ALAT: Alanine amino transferase
DST: Digit symbol test
HE: Hepatic encephalopathy
INR: International normalized ratio
LTT: Line tracing test
LTTe: Line tracing test error
LTTt: Line tracing test time
MELD: Model for end stage liver disease
MHE: Minimal hepatic encephalopathy
NCT-A: Number connection test-A
NCT-B: Number connection test-B
PHES: Psychometric hepatic encephalopathy score
PSE: Porto systemic encephalopathy
SD: Standard deviation
SDT: Serial dotting test
SPSS: Statistical package for social sciences
