# Supplementary material for: Normalisation of the psychometric encephalopathy score within the Cameroonian population
Source: BMC Gastroenterol. 2021 Jul 12;21:287. doi: 10.1186/s12876-021-01858-7 (PMC8273953; doi:10.1186/s12876-021-01858-7)
Supplement: Supplementary file 1 — Additional file 1. Example of calculation of the Z score for each test and the PHES of a cirrhotic patient. [file 12876_2021_1858_MOESM1_ESM.docx]

**Example of calculation of the Z score for each test and of the PHES of a cirrhotic patient.**

**Cirrhotic patient characteristics:**

Age: 36 Years

Education: 16 years

**Scores (collected variables):**

NCT-A: 56 NCT-B: 61 SDT: 94 LTT: 117 DST: 36

**Calculation of each score test with equation of predictors of psychometric tests on multivariate analysis for cirrhotics.**

| Test | Equation |
| --- | --- |
| NCT-A | 234.67 +2.2×age – 18.58×education |
| NCT-B | 369.25 +2.98×age – 27.07×education |
| SDT | 195.60 + 1.12×age - 10.46×education |
| LTT | 215.25 + 1.05×age - 8.73×education |
| DST | 16.89 – 0.21×age + 1.7×education |

**Calculated results:**

NCT-A: 16.59 NCT-B: 43.41 SDT: 68.56 LTT: 113.37 DST: 36.53

**Correspondence of each test result with corresponding** **Z score established with healthy participants performances.**

NCT-A: 1 NCT-B: 1 SDT: 0 LTT: 0 DST: 0

**Total (PHES score): 2**

**Conclusion: Absence of minimal hepatic encephalopathy.**

**Z score values established with healthy participants performances.**

**Principles of interpretation**

| **DST (Points)** | | **NCT-A, NCT-B, SDT and LTT (Seconds)** | |
| --- | --- | --- | --- |
| > Mean + 1 SD | +1 point | > Mean + 3 SD | -3 points |
| ] Mean - 1 SD ; mean + 1 SD] | 0 point | ] Mean +2 and mean +3 SD] | -2 points |
| ] Mean -1 and mean -2 SD] | -1 point | ] Mean +1 and mean +2 SD] | -1 point |
| ] Mean -2 and mean -3 SD] | -2 points | ] Mean - 1 SD ; mean + 1 SD] | 0 point |
| ≤ Mean -3 SD | -3 points | ≤ Mean - 1 SD | +1 point |

**DST Score (points)**

| M-3 SD | M-2 SD | M-1 SD | Moyenne | M+1 SD | SD |
| --- | --- | --- | --- | --- | --- |
| - 31.17 | - 20.78 | -10.39 | 34.47 | +10.39 | 10.39 |
| 3.30 | 13.69 | 24.08 | 34.47 | 44.86 |  |

> 44.86 : +1 point

]24.08 ;44.86] : 0 point

]13.69 and 24.08] : -1 point

]3.30 and 13.69] : -2 points

≤ 3.30 : -3 points

**NCT-A Score**

| M-1 SD | Mean | M+1 SD | M+2 SD | M+3 SD | SD |
| --- | --- | --- | --- | --- | --- |
| -30.78 | 85.14 | +30.78 | +61.56 | +92.34 | 30.78 |
| 54.36 | 85.14 | 115.92 | 146.7 | 177.48 |  |

>177.48 : -3 points

]146.70 and 177.48] : -2 points

]115.92 and 146.7] : -1 point

]54.36 ; 115.92] : 0 point

≤ 54.36 : +1 point

**NCT-B Score**

| M-1 SD | Mean | M+1 SD | M+2 SD | M+3 SD | SD |
| --- | --- | --- | --- | --- | --- |
| -64.63 | 120.33 | +64.63 | +129.26 | +193.89 | 64.63 |
| 55.70 | 120.33 | 184.33 | 249.59 | 314.22 |  |

>314.22 : -3 points

]249.59 and 314.22] : -2 points

]184.33 and 249.59] : -1 point

]55.70 ; 184.33] : 0 point

≤ 55.70 : +1 point

**SDT Score**

| M-1 SD | Mean | M+1 SD | M+2 SD | M+3 SD | SD |
| --- | --- | --- | --- | --- | --- |
| -20.55 | 73.56 | +20.55 | +41.10 | +61.65 | 20.55 |
| 53.01 | 73.56 | 94.12 | 114.66 | 135.21 |  |

> 135.25 : -3 points

]114.66 and 135.25] : -2 points

]94.12 and 114.66] : -1 point

]53.01 ; 94.12] : 0 point

≤ 53.01 : +1 point

**LTT Score**

| M-1 SD | Mean | M+1 SD | M+2 SD | M+3 SD | SD |
| --- | --- | --- | --- | --- | --- |
| -33.92 | 109.83 | +33.92 | +67.84 | +101.76 | 33.92 |
| 75.91 | 109.83 | 143.75 | 177.67 | 211.59 |  |

>211.59: -3 points

]177.67 and 211.59] : -2 points

]143.75 and 177.67] : -1 point

]75.91 ; 143.75] : 0 point

≤ 75.91 : +1 point

**PHES Score**

- Sum of NCT-A, NCT-B, SDT, LTT and DST points
- Total value between -15 and +5 points
- Presence of minimal hepatic encephalopathy if score <-3.

**Abbreviations:**

DST: Digit Symbol Test

LTT: Line Tracing Test

M: Mean

NCT-A: Number Connection Test A

NCT-B: Number Connection Test B

PHES: Psychometric Hepatic Encephalopathy Score

SD: Standard Deviation

SDT: Serial Dotting Test
